# Knockout of *ho-1* protects the striatum from ferrous iron-induced injury in a male-specific manner in mice

**DOI:** 10.1038/srep26358

**Published:** 2016-05-20

**Authors:** Li-Fang Wang, Kazunari K. Yokoyama, Chih-Lung Lin, Tzu-Yin Chen, Hsiu-Wen Hsiao, Pei-Chi Chiang, Chin Hsu

**Affiliations:** 1 Department of Medicinal and Applied Chemistry, College of Life Science, Kaohsiung Medical University, No. 100, Shih-Chuan 1st Road, Kaohsiung, Taiwan; 2 Graduate Institute of Medicine, Kaohsiung Medical University, No. 100, Shih-Chuan 1st Road, Kaohsiung, Taiwan; 3 Department of Neurosurgery, Faculty of Medicine, College of Medicine; Kaohsiung Medical University, No. 100, Shih-Chuan 1st Road, Kaohsiung, Taiwan; 4 Department of Physiology, Faculty of Medicine, College of Medicine; Kaohsiung Medical University, Kaohsiung, No. 100, Shih-Chuan 1st Road, Kaohsiung, Taiwan

## Abstract

Men have worse survival than premenopausal women after intracerebral hemorrhage (ICH). After ICH, overproduction of iron associated with induction of heme oxygenase-1 (HO-1) in brain was observed. Rodent ICH model using ferrous citrate (FC)-infusion into the striatum to simulate iron overload, showed a higher degree of injury severity in males than in females. However, the participation of HO-1 in sex-differences of iron-induced brain injury remains unknown. The present results showed a higher level of HO-1 expression associated with more severe injury in males compared with females after FC-infusion. Estradiol (E_2_) contributed to lower levels of FC-induced HO-1 expression in females compared with males. Heterozygote *ho-1* KO decreased the levels of FC-induced injury severity, histological lesions, behavioral deficits, autophagy and autophagic cell death in the striatum of males but not in females. Moreover, *ho-1* deficiency enhanced the neuroprotection by E_2_ only in males. These results suggested that over induction of HO-1 plays a harmful role in FC-induced brain injury in a male-specific manner. Suppression of HO-1 combined with E_2_ exhibits a synergistic effect on neuroprotection against FC-induced striatal injury in males. These findings open up the prospect for male-specific neuroprotection targeting HO-1 suppression for patients suffering from striatal iron overload.

Intracerebral hemorrhage (ICH) is a common subtype and accounts for approximately 10% to 20% of strokes^1^. It has the highest mortality in all stroke types and a higher mortality in men than in premenopausal women^2^,^3^. Previous studies indicated that men and women respond differently to stroke treatment^4^,^5^. However, the recommendation for treatment after hemorrhagic stroke in men and women appears to be the same^6^. Thus, understanding sex dimorphism in host responses to stroke insult may help to developing sex specific therapeutic strategies for those patients suffering from brain functional deficits after hemorrhagic stroke.

Sex differences are ubiquitous in neurological disorders. The striatum is the area where ICH commonly occurs^7^. After hemorrhage, blood leaks into the brain parenchyma and the accumulation of iron and subsequent free radical generation contributes to neuronal loss and neurological deficits^8^,^9^. In the meantime, oxidative stress challenges HO-1 expression and subsequently increases the heme catabolism into biliverdin and bilirubin, which are potent antioxidants^10^,^11^. Induction of HO-1 may protect cells by scavenging free radicals^12^, whereas HO-1 induction was associated with a deleterious iron accumulation in activated microglia in a rodent stroke model^13^. A recent study showed that HO-1 deletion reduces iron deposition/toxicity in a mouse model of ICH^14^. Thus, the role of HO-1 in sex dimorphism of brain injury caused by iron overload remains uncertain. Because male mice brains were more sensitive (by 3–4-fold) than female brains to oxidative stress^15^ and men exhibit worse free radical homeostasis and weaker defense capacities against oxidative stress than women^16^, the inducible HO-1 may respond to iron overload in a sex-dimorphic manner. The present study was designed to test the hypothesis that ferrous iron causes HO-1 over induction and suppression of HO-1 protects the striatum from iron overload in a male-specific manner.

## Results

### Sex differences in the FC-induced HO-1 expression and striatal injury

**Figure 1:**
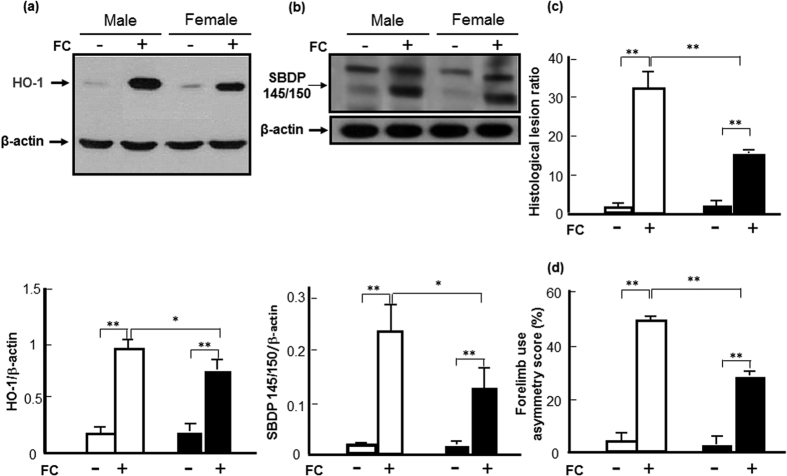
Sex differences in the levels of HO-1, injury severity, histological lesions and behavioral deficits after ferrous citrate (FC)-infusion. (**a**) Protein level of HO-1. White column: male; black column: female.(**b**) Level of SBDP 145/150. (**c**) Histological lesion ratio. (**d**) Forelimb use asymmetry score. Data are expressed as the mean ± SD (n = 6), *indicates P < 0.05, **indicates P < 0.01.

To know whether there is a correlation between the level of HO-1 induction and the sex-differences of striatal injury caused by iron overload, the levels of HO-1, injury severity, histological lesions and behavioral deficits were evaluated in both male and female mice after FC-infusion. The results showed that FC infusion increased the HO-1 protein levels in the striata of male and female mice by 4.9-fold and 3. 5-fold, respectively (Fig. 1a). With a similar trend, FC infusion increased the levels of SBDP 145/150, which depicts injury severity, by 8.8-fold vs 4.0-fold (Fig. 1b), the lesion ratio by 16.3-fold vs 7.9-fold (Fig. 1c) and the forelimb use asymmetry score by 9.6-fold vs 7.4-fold (Fig. 1d) in male vs female mice, respectively. The iron deposition, which is confirmed by Prussian blue assay, after FC infusion in the striatum of male mouse was more than that in female as shown in the supplementary information (Fig. S1). The images by HE stain showed that the histological lesion in the striatum of male was also severer than that of female after FC infusion as shown in the supplementary information (Fig. S2).

### Role of E_2_ in sex differences of HO-1 induction and spectrin cleavage after FC infusion

**Figure 2:**
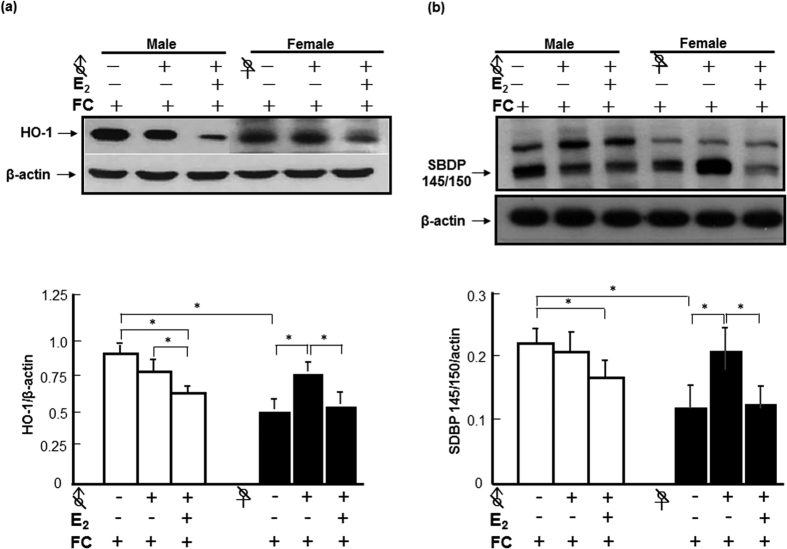
E_2_ contributes to the sex differences in HO-1 induction and injury severity after FC-infusion. ♂ or ♀ with a slash indicated orchidectomy or Ovariectomy, respectively. White column: male; black column: female. (**a**) Protein level of HO-1. (**b**) Level of SBDP 145/150. The data are expressed as the mean ± SD (n = 6), *indicates p < 0.05.

To examine whether endogenous E_2_ contributes to the lower levels of FC-induced HO-1 expression and injury severity in females, the levels of HO-1 and cleavage of α-II spectrin were compared between FC-infused castrated mice with and without E_2_ implantation. The results showed that orchiectomy did not change the level of HO-1 induction by FC in males, but ovariectomy increased the level of HO-1 induction by FC infusion in females. Orchiectomy diminished the sex-differences in FC-induced HO-1 expression and injury severity. E_2_ implantation decreased the protein levels of HO-1 in castrated males and ovariectomized females after FC-infusion (Fig. 2a). Similarly, orchiectomy did not change the level of FC-induced cleavage of spectrin in males, but increased the level of FC-induced cleavage of spectrin in female mice. E_2_ implantation decreased the levels of FC-induced cleavage of spectrin to SBDP 145/150 in both castrated males and females (Fig. 2b).

**Figure 3:**
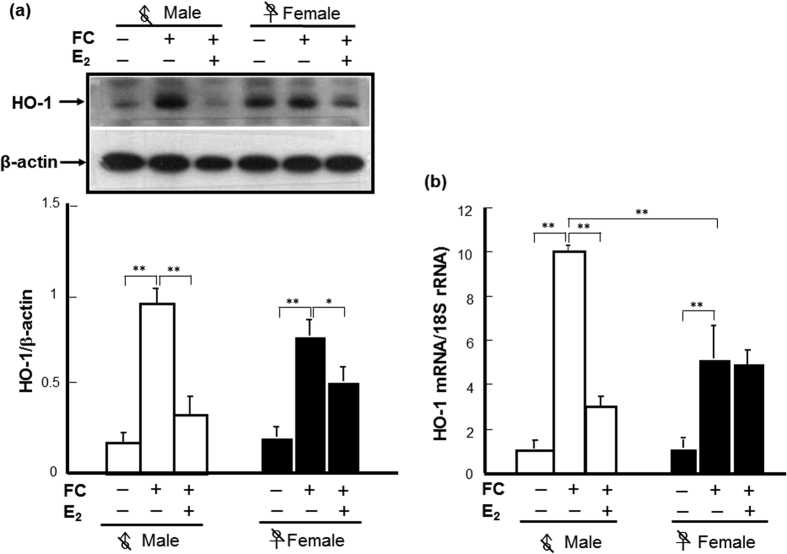
E_2_ decreased the FC-induced HO-1 expression at both levels of protein and mRNA in castrated male mice. (**a**) Protein levels of HO-1. (**b**) Levels of HO-1 mRNA. The data are expressed as the mean ± SD (N = 6). *indicates p < 0.05; **indicates p < 0.01.

To address whether E_2_ inhibits the FC-induced HO-1 expression at a translational level or transcriptional level, the levels of mRNA and protein were compared between castrated mice with and without E_2_ implantation. The results showed that, after orchiectomy/ovariectomy, no sex difference in protein or mRNA levels of HO-1 induction by FC-infusion was observed. In castrated males, exogenous E_2_ decreased the levels of HO-1 protein and mRNA by 67.7% and 69.9%, respectively. In ovariectomized females, E_2_ decreased the HO-1 protein level by 35.0% and mRNA by 7.7% (Fig. 3a,b).

### Effect of *ho-1* deficiency on FC-induced striatal injury and autophagy

**Figure 4:**
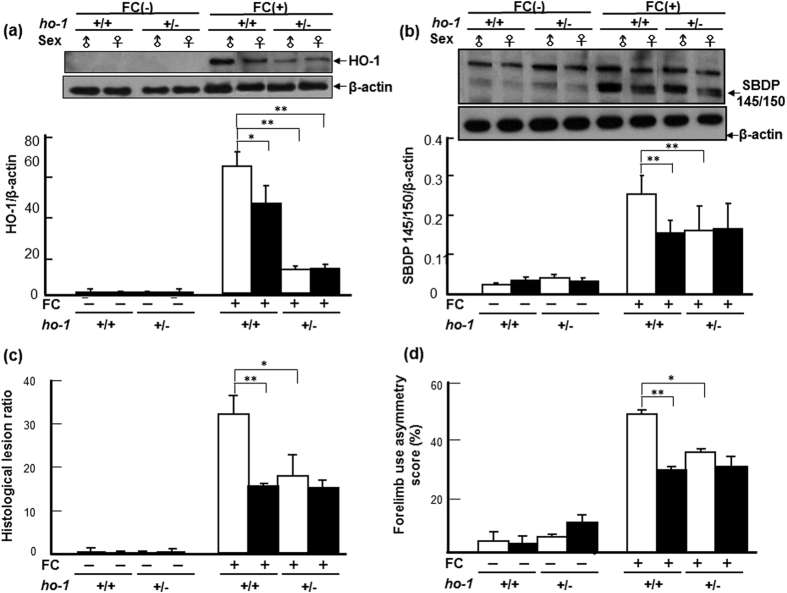
Heterozygous KO of *ho-1* decreased the severity of FC-induced striatal injury in a male-specific manner. (**a**) Protein level of HO-1. (**b**) Level of SBDP 145/150. (**c**) Histological lesion ratio. (**d**) Forelimb use asymmetry score. The data are expressed as the mean ± SD (n = 6). *indicates p < 0.05; **indicates p < 0.01.

As shown in Fig. 4a, the levels of HO-1 in both male and female *ho-1*^+/−^ mice were significantly lower than those in *ho-1*^+/+^ mice, respectively. Similar to C57BL/6 mice, FC-induced a higher level of HO-1 in male than in female *ho-1*^+/+^ mice. While no sex-difference in FC-induced HO-1 expression in *ho-1*^+/−^ mice was observed (Fig. 4a).

To elucidate the effect of heterozygote *ho-1* KO on FC-induced striatal injury, cleavage of α-II spectrin, histological lesions, and behavioral deficits were compared between *ho-1*^+/−^ and *ho-1*^+/+^ mice after FC infusion. Similar to the result from C57B/6 mice, FC infusion caused higher levels of SBDP 145/150 (Fig. 4b), lesion ratios (Fig. 4c), and forelimb use asymmetry scores (Fig. 4d) in *ho-1*^+/+^ males than those in *ho-1*^+/+^ females. Heterozygous KO of the *ho-1* gene decreased the levels of FC-induced spectrin cleavage, histological lesions, and behavioral deficits in male but not in female mice, and diminished the sex differences in FC-induced striatal injury (Fig. 4b–d).

**Figure 5:**
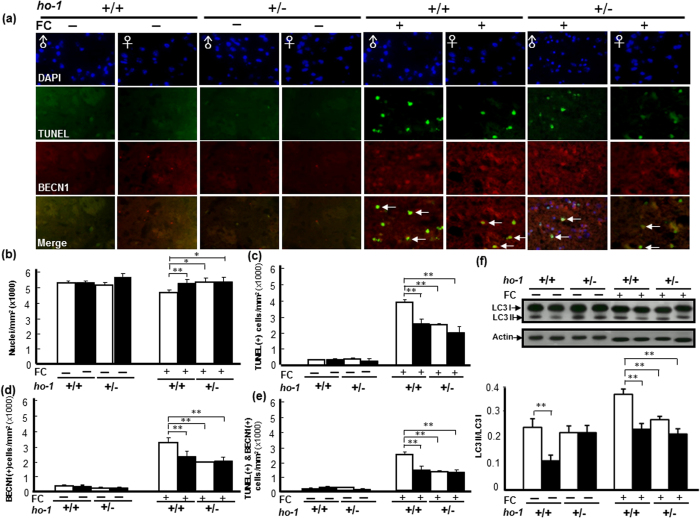
Male specific alleviation of FC-induced autophagic cell death and decrease of autophagy in the striatum by *ho-1* KO mice. (**a**) Representative images of TUNEL(+) BECN1 immunoreactive cells. (**b**) Number of nuclei. (**c**) TUNEL(+) cells. (**d**) BECN1 immunoreactive cells. (**e**) TUNEL(+) BECN1 immunoreactive cells. (**f**) Level of autophagy. The data are expressed as the mean ± SD (n = 6). *indicates p < 0.05, **indicates p < 0.01.

We next investigated whether heterozygote *ho-1* KO affected the FC-induced autophagy and autophagic cell death, the level of LC3-II and the number of TUNEL(+) BECN1 immunoreactive cells were compared between striata of *ho-1*^+/−^ and *ho-1*^+/+^ mice after FC-infusion, respectively. The results showed that heterozygous KO of the *ho-1* gene decreased both BECN1 immunoreactivity and the number of TUNEL(+) cells in the striatum of male mice with FC infusion (Fig. 5a). The representative images were taken in the enclosed areas (striata) as indicated in the supplementary information (Fig. S2). The quantitative result showed that, without FC-infusion, heterozygous KO of the *ho-1* gene did not change the number of nuclei in both *male and female* mice. FC-infusion decreased the number of nuclei in males and heterozygous KO of *ho-1* gene diminished the FC-induced nucleus loss in males but not in females (Fig. 5b). FC-infusion induced higher levels of DNA fragmentation (TUNEL positive), BECN1 immunoreactivity, and TUNEL(+) BECN1 immunoreactivity in males than in females (Fig. 5c–e). Heterozygous KO of the *ho-1* gene decreased the number of TUNEL(+) cells, BECN1 immunoreactive cells and TUNEL(+) BECN1 immunoreactive cells after FC-infusion in males, but not in females. Similarly, FC-infusion caused a higher ratio of LC3-II to LC3-I, which depicts level of autophagy in males compared to females, whereas heterozygote KO of *ho-1* gene decreased the level of FC-induced autophagy in males but not in females. Interestingly, without FC-infusion, the basal level of LC3-II was higher in males than in females and heterozygote *ho-1* KO diminished the sex-differences in the level of constitutive autophagy in intact mice (Fig. 5f).

### Heterozygote KO of *ho-1* gene enhanced the neuroprotective effect of E_2_ against FC-induced striatal injury in a male-specific manner

**Figure 6:**
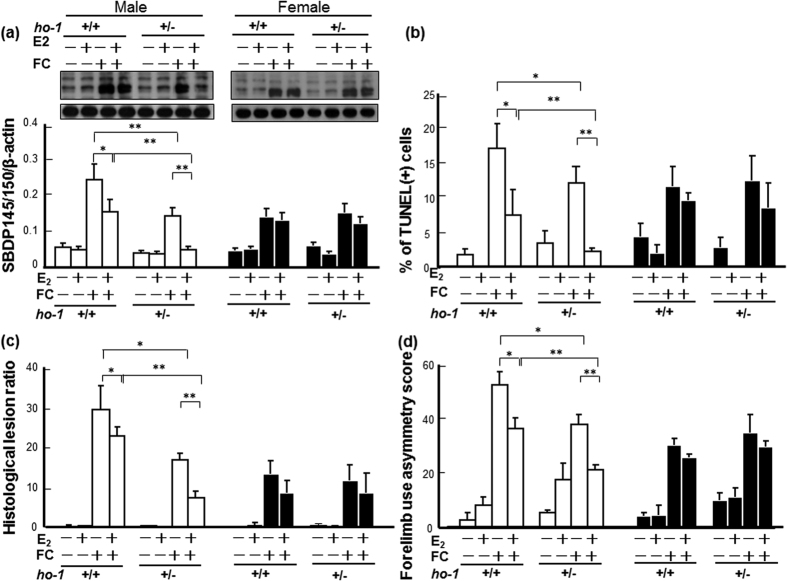
Heterozygous KO of *ho-1* enhanced the neuroprotective effect of E_2_ against FC-induced striatal injury in male mice. (**a**) Level of SBDP 145/150. (**b**) Number of TUNEL(+) cells. (**c**) Histological lesion ratio. (**d**) Forelimb use asymmetry score. The data are expressed as the mean ± SD (n = 6). *indicates p < 0.05; **indicates p < 0.01.

In order to know whether HO-1 suppression mediates the neuroprotective effect of E_2_ against FC-induced striatal injury, the protective effects of E_2_ on FC-induced striatal injury were compared between *ho-1*^+/−^ and *ho-1*^+/+^ mice. The results showed that *ho-1* deficiency *per se* simulates the protective effect of E_2_ on FC-induced striatal injury in males but not in females. Moreover, the neuroprotective effect of E_2_ in *ho-1*^+/−^ males was better than that in *ho-1*^+/+^ males. However, no significant difference in neuroprotection by E_2_ between *ho-1*^+/−^ and *ho-1*^+/−^ was observed in females. As shown in Fig. 6a–d, in males, E_2_-treatment decreased the FC-induced spectrin cleavage, behavioral deficits, lesions, and DNA fragmentation in the *ho-1*^+/+^ group by 33.3%, 30.8%, 23.3%, 55.9%, respectively, and by 65.9%, 46.9%, 56.6%, 82.1%, in the *ho-1*^+/−^ group, respectively. In females, E_2_-treatment decreased the FC-induced spectrin cleavage, behavioral deficits, lesions, and DNA fragmentation in the *ho-1*^+/+^ group by 7.1%, 13.3%, 30.8%, 25.0%, respectively, and by 16.7%, 14.3%, 25.5%, 40.0% in the *ho-1*^+/−^ group, respectively.

## Discussion

The present study demonstrated that a higher level of HO-1 associated with higher levels of injury severity, histological lesions and behavioral deficits in males than in females was induced by striatal FC-infusion, which simulates iron overload in the striatum after ICH. E_2_ contributes to the lower levels of FC-induced HO-1 expression and injury severity in females than in males. Heterozygote KO *ho-1* gene diminished the FC-induced striatal injury in males but not in females. In addition, heterozygote KO *ho-1* gene simulates and enhances the neuroprotective effect of E_2_ on FC-induced striatal injury. These results suggested that the high level of FC-induced HO-1 in males may exaggerate the FC-induced striatal injury, therefore HO-1 suppression diminished the FC-induced striatal injury and favored the outcome of males after iron overload. Our study, for the first time, explored the sex dimorphic effect of HO-1 suppression on the FC-induced striatal injury. These findings open up the prospect for a male-specific neuroprotection targeting HO-1 suppression for patients suffering from striatal iron overload.

Understanding of the sex difference in neurological recovery and responses to therapeutics may help the successive preclinical translation. There is growing literature examining sex differences in stroke epidemiology, however conflicting evidences of sex differences in outcome exist after stroke. Previous study reported that, without examining the effect of sex by stroke subtype, women aged 55 to 84 years had lower mortality than men, while women aged more than 85 years had 15% higher stroke mortality than men^2^. Another report indicated that, for younger patients after ICH, female sex was protective; however, at ages greater than 60 years, female sex was a risk factor for discharge to hospice or death. Their statistical analysis about the interaction between gender and age demonstrated significantly strong correlation with early outcome after ICH^17^. On the other hand, Dehlendorff *et al*. reported that, after the age of 60 years, elderly women were affected more severely than men with ischemic stroke but not with hemorrhagic stroke^18^. Recently, the report from heart disease and stroke statistics-2016 updates indicated that women have greater levels of disability than men after stroke^19^. Usually, women are older than men when they have a stroke, and severity increases with age. Moreover, the numbers of older women were higher than men in the population, the clinical trial enrollment with analyzing stroke types or age factor separately may decrease the inconsistency in the sex-difference in the severity after stroke. In the present study, we used FC-infusion mouse model to simulate accumulation of ferrous iron after hemorrhage. The result showed that FC-infusion induced a higher degree of striatal injury in male mice than that in age-matched females indicating FC-infusion model simplifies the detrimental factor and excludes the effect of age factor after hemorrhagic stroke. This model is proper for studying sex-dimorphic striatal injury due to iron overload that simulates hemorrhagic stroke.

The *ho-1* gene is exquisitely sensitive to induction by a wide range of pro-oxidant and other stressors including ICH^20^. After hemorrhage, clot lysis and iron resulting from hemoglobin degradation play important roles in ICH-induced brain injury^21^. Brain iron accumulation may induce neuronal damage even after it has become bound to ferritin because iron can be released in its ferrous form under the acidic conditions^22^. Iron toxicity is largely based on Fenton chemistry where iron reacts with reactive oxygen intermediates to produce highly reactive free radical species such as the hydroxyl radical (OH^*−*^)^23^. A previous report indicated that deferoxamine (an iron chelator) reduced the number of HO-1 (p < 0.01) positive cells in the ipsilateral basal ganglia in a rat model of ICH by intracaudate injection of autologous blood^24^, suggesting iron overload contributes to the HO-1 induction after ICH. In the present study, castration diminished the sex-difference in FC-induced HO-1 expression. Moreover, castration increased, while E_2_ implantation decreased, the levels of HO-1 induction (Fig. 2a). This suggested that the suppressive effect of E_2_ on HO-1 induction contributes to the lower level of HO-1 induction associated with injury severity caused by FC-infusion in females compared with males.

The effect and mechanism underlying the effect of E_2_ on HO-1 induction remains controversial. A previous report indicated that E_2_ treatment led to Nrf2 dissociation from Keap1 in the cytoplasm, and then translocated into nucleus, with a significant increase in HO-1 expression in homocysteine-treated cells^25^. Another study demonstrated that the protective effects of E_2_ in male rats were ERα-independent and might be associated with HO-1 inhibition^26^. In the present results, E_2_ significantly inhibited HO-1 induction by FC infusion in the striatum of male mice at both levels of mRNA and protein, while E_2_ inhibited FC-induced HO-1 expression at the level of protein but not at an mRNA level in females (Fig. 3). This result suggested an ERα-independent mechanism underlying the suppression of HO-1 expression by E_2_ in the striatum of male mice, because the expression level of ERα mRNA and protein and the number of ERα immunoreactive cells in the striatum were higher in female than in male brains^27^. However, further investigation is needed to understand how E_2_ decreases FC-induced HO-1 expression.

HO-1 is an enzyme exhibiting both beneficial and harmful effects, depending on the different experimental models^28^. Its beneficial effects have been related to the catabolism of the prooxidant heme to biliverdin and bilirubin, which are potent antioxidants^29^,^30^, if produced in excess, unconjugated bilirubin becomes neurotoxic through multiple mechanisms involving the disruption of cell membrane structure, the reduction of mitochondrial transmembrane potential and the activation of the apoptotic cascade^31^,^32^. While a massive HO-1 activation may metabolize heme into free iron protoporphyrin, it then releases equimolar amounts of ferrous iron^33^,^34^, which catalyzes reactions that generate ROS. Iron chelators decreased microglia activation in the ipsilateral hemisphere after hemin and quinolinic acid injection, strongly suggesting that Fe^2+^ may be implicated in deleterious HO-1 effects^35^. Previous reports indicated that a HO-1 inhibitor, SnPP, attenuated edema in a hemoglobin-induced model of ICH^36^ and HO-1 inhibition by tin-mesoporphyrin IX (SnMP) protection against neuronal loss in a rabbit model of autologous blood injection^37^. In addition, SnMP-treatment also reduced edema development following experimental ICH in pigs^38^. These results suggest a harmful role of HO-1 in hemorrhagic stroke. Moreover, deletion of the *ho-1* gene results in diminution of brain tissue injury in a male mouse model of ICH by collagenase injection in the caudate putamen^39^. In the hemorrhagic brain of mice, HO-1 expression is mainly observed in microglia/macrophages around the hematoma region. Notably, *ho-1* deletion is also associated with a decreased number of activated microglia/macrophages in thrombin-induced brain injury, suggesting that HO-1 supports activated microglia survival, which may exacerbate striatal injury^40^. In the present results, a significantly higher level of FC-induced HO-1 expression in the striatum was associated with a higher severity of striatal injury of intact males than that in intact females after FC-infusion (Fig. 1a). Furthermore, heterozygote KO *ho-1* gene diminished the FC-induced striatal injury as well as the FC-induced autophagy and autophagic cell death in males but not in females, implicating a harmful role for massive HO-1 induction in males. While the moderate level of HO-1 induction after FC-infusion in females may help with hormesis under a redox microenvironment.

Basal HO-1 expression is maintained at a low level in the normal brain and is restricted to small groups of scattered neurons and neuroglia^12^. Induction of glial *ho-1* may lead to the accumulation of iron^41^, which may exacerbate intracellular oxidative stress and subsequent injury by promoting free radical generation within^42^. The activity of heme oxygenase has also been shown to enhance vulnerability in various experimental models of neural injury and disease^43^,^39^,^44^. A previous report indicated that over-expression of the *ho-1* gene promotes mitochondrial membrane damage, unregulated iron deposition, and macroautophagy in cultured astroglia^45^. Our recent report showed that iron-induced striatal injury is alleviated by inhibition of autophagy in a male-specific manner^46^. Whether the deleterious HO-1 activation effect in males on iron toxicity is linked to autophagy remains unknown. In the present study, we examined whether autophagy inhibition mediates the protective effect of *ho-1*deficiency on FC-induced striatal injury. Interestingly, we found that, without FC-infusion, the basal level of constitutive autophagy was higher in males than in females and *ho-1*deficiency diminished the sex-difference in the level of constitutive autophagy (Fig. 5f). This result suggested that the suppressive effect of E_2_ on the basal level of autophagy is HO-1 dependent. On the other hand, after FC-infusion, both the amount of autophagic cell death and the level of LC3-II in *ho-1*^+/+^ males were higher than those in *ho-1*^+/−^ males, respectively. Although the interaction between HO-1 and autophagy remained obscure, these results implied that HO-1 may enhance vulnerability in FC-induced autophagy and autophagic cell death. Therefore, *ho-1* deficiency conferred protection, at least partially, by diminishing the FC-induced autophagy and autophagic cell death.

Estrogen is both a natural neuroprotectant and a potential therapeutic agent for cerebrovascular disease. Pretreatment with E_2_ attenuates brain edema after ICH in male mice^47^. In addition, estrogen reduces iron-induced brain edema *in vivo* and neuronal death *in vitro*^48^. Several mechanisms may contribute to the beneficial effect of estrogen on brain injury. A previous report showed that administration of E_2_ to male (but not female) rats significantly reduced brain edema, neurological deficits, and the level of HO-1 induced by ICH^26^,^49^. This effect of E_2_ in males might be ER-α-independent and might be associated with HO-1 inhibition^26^. Another report indicated that E_2_ protects against light-induced retinal damage via its antioxidative effect^50^. In the present results, no significant effect of *ho-1* deficiency on E_2_ neuroprotection was observed in the female group (Fig. 6). While, in male mice, *ho-1* deficiency per se slightly simulated the protective effect of E_2_ on FC-induced striatal injury, suggesting HO-1 suppression partially mediates the neuroprotective effect of E_2_. Moreover, *ho-1* deficiency further enhanced the protective effect of E_2_ against FC-induced striatal injury in males but not in females, implicating the synergistic protection of HO-1 suppression with E_2_ may be ERα-independent because the level of ERα in the striatum was higher in females than in males.

In conclusion, HO-1 plays a sex dimorphic role in FC-induced brain injury and HO-1 silencing has a sex-specific benefit for iron-induced striatal injury only in males because they lack the endogenous protection conferred by estradiol. These findings open up the prospect for a male-specific injury prevention targeting HO-1 inhibition for patients suffering from acute iron overload caused by ICH.

## Materials and Methods

### Animals

A total of 120 age-matched (12-week-old) male (♂) and female (♀) C57BL/6 mice [purchased from the National Laboratory Animal Center, Taipei, Taiwan] were used for studying the sex-differences in FC-induced HO-1 expression and injury severity. To study the contribution of E_2_ to the sex difference in FC-induced HO-1 expression in the striatum, C57BL/6 mice implanted with estradiol (E_2_) after castration were used. For female mice, ovariectomy involves a small incision in both flanks after which the ovaries are removed. In male mice, the skin and body wall caudal to the penis were incised with a scissor, and the testicles were retracted through the incision. The exposed testicular artery and vein were double-ligated and cut^51^. A silastic tube (2 mm inner diameter, 20 mm in length) containing 0.38 mM E_2_ (Sigma-Aldrich, E8515) was implanted subcutaneously 24 h before FC infusion. Three μl of FC (1 nmol/μl) (Sigma-Aldrich, St. Louis, USA Sigma) were infused into the right striatum (coordinates: 0.2 mm anterior, 2.5 mm lateral, and 3.5 mm ventral to the bregma) using a microinfusion pump [CMA Microdialysis] at a rate of 1 μl/min. Sham-operated mice were used as the control group. Two days after FC infusion, the forelimb use asymmetry test was performed to evaluate the FC-induced behavioral deficits. Then, the brain tissue containing the striatum was sampled and frozen for western blot analysis or was fixed and sectioned in 10 μm thicknesses for the evaluation of histological lesions or immunohistochemical detection. In addition, a total of 144 age-matched (12-week-old) male (♂) and female (♀) heterozygous *ho-1* KO (*ho-1*^+/−^) mice [breeding pairs of *ho-1* heterozygous mice was derived from Dr. Shaw-Fang Yet, Harvard University via Brigham and Women’s Hospital, Boston, MA, USA] were used for investigating the role of HO-1 in sex differences of FC-induced striatal injury. Heterozygous *ho-1* KO (*ho-1*^+/−^) mice were used because *ho-1*^−/−^ mice were neonatally lethal. No animals were excluded. All operations were performed under anesthesia with Zoletil 50 (1 ml/kg B.W. intraperitoneally) and were approved by the Kaohsiung Medical University Committee for the Use of Experimental Animals. All experiments were performed in accordance with the approved guidelines.

### Western blot analysis

Brain tissue containing the striatum was sampled and homogenized in 100 μL lysis buffer (50 mM Tris acetate, pH 7.4, 5 mM ethylenediamine tetraacetic acid) and then, was centrifuged at 14,000 r.p.m for 30 minutes. The protein concentration of the supernatant was determined with a protein assay kit [Bio-Rad Corp., Hercules, CA]. An equal amount of protein from each sample was separated by sodium dodecyl sulfate-polyacrylamide gel electrophoresis (SDS-PAGE), transferred onto a PVDF membrane (NEN Life Science Products, Boston, USA), incubated with mouse antibodies against HO-1 monoclonal antibodies (1:2,000, ADI-OSA-110, Stress-Gen Biotechnologies), mouse α-II spectrin antibodies (1:1,000, sc-7465, Santa Cruz Laboratories), rabbit anti-LC3 (microtubule associated protein light chain 3) antibodies (1:2,000, L-7543, Sigma), or mouse anti-β-actin (1:5,000) (Chemicon, CA, USA) followed by goat anti-rabbit second antibodies (1: 5,000 in t-TBS/5% milk) (Santa Cruz Laboratories, Santa Cruz, CA) or goat anti-mouse secondary antibodies (1:10,000 in TBS-t/5% milk) (Jackson ImmunoResearch Laboratories, Inc., West Grove, USA) at room temperature for 1 hour, and then visualized by ECL chemiluminescence (NEL 105, PerkinElmer Life and Analytical Science) and subsequently exposed to X-ray film^52^. The ratio of LC3-II to LC3-I depicts the level of autophagy. The level of SBDP 145/150 that was normalized by actin acts as an index of severity of injury.

### Quantification of histological lesions

The paraffin-embedded tissue was serially sectioned into sections of 10 μm in thickness. After hematoxylin and eosin (H&E) staining, the level of histological lesions in every fifteenth section containing the striatum was analyzed using Image-pro Plus software (Universal Imaging Corp.) according to the staining intensity of the striatal area. The histological lesion ratio was estimated by dividing the hemispheric volume of the striatum on the ipsilateral side by that on the contralateral side. The iron accumulation was confirmed by Prussian blue assay on brain sections from mice infused with FC.

### Forelimb use asymmetry test

An individual mouse was placed in a transparent cylinder (10 cm in diameter and 15 cm in height) in the dark, and the use of ipsilateral limbs (I), contralateral limbs (C), or simultaneous use of both forelimbs (B) was observed for a 5-min period. The test was randomized, blinded, and repeated twice in each mouse. The forelimb use asymmetry score was calculated using the following equation: [I/(I + C + B)] −[C/(I + C +B )]^53^.

### Real-time polymerase chain reaction

Total RNA from brain tissue containing the striatum was extracted using TRIzol reagent (Invitrogen, Carlsbad, CA). Complementary DNA was transcribed at 42 °C for 60 min using SuperScript II RNase H-Reverse Transcriptase with oligo and random primers (Promega Corporation, Madison, Wis) in a reaction mixture containing 50 mM Tris-HCl, 50 mM KCl, 10 mM MgCl2, 10 mM dithiothreitol, 0.5 mM spermidine, and 1 mM deoxynucleotide triphosphates. Primers for HO-1 and 18S ribosomal RNA were designed as the following sequences, respectively: 5′-AGGTGTCCAGGGAAGGCTTTA-3′ (forward) and 5′-TAATGCCTTCCCTGGACACCT-3′ (reverse) and 5′-CGCAGCTAGGAATAATGGAATAGG-3′ (forward) and 5′-CATGGCCTCAGTTCCGAAA-3′ (reverse). Real-time PCR was performed in a 25 μL reaction mixture containing 12.5 μL of 2x SYBR Green PCR Master Mix (Applied Biosystems), 50 ng complementary DNA, and either 300 nM primers for HO-1 or 100 nM primers for 18S rRNA. Samples were analyzed in triplicate using the following thermocycling conditions: 50 °C for 2 min and 95 °C for 10 min, denaturation at 95 °C for 15 s, annealing at 60 °C for 1 min for 40 amplification cycles in total.

### Immunostaining and TUNEL staining

The tissue sections were deparaffinized and rehydrated by immersing in xylene followed by a series of immersions in graded ethanol and double distilled water. The sections were then washed in 0.1 M PBS for 10 min and incubated for 30 min at 37 °C with proteinase K solution, nuclease free treatment, followed by quenching endogenous peroxidase with 3% H_2_O_2_ in methanol for 20 min. For antigen retrieval, the sections were placed in 0.1 M citrate acid (pH 6.0) and irradiated with 350 W microwaves for 5 mins. Then sections were subsequently incubated with blocking solution containing 2% Bovine Serum Albumin (BSA, Sigma chemical CO.), 10% Normal Goat Serum (NGS, Jackson ImmunoResearch Laboratories, Inc., West Grove, USA) and 1% Triton X-100 for 30 mins at room temperature. Mouse anti-BECN1 antibodies (1:50; #612112, BD Biosciences) were used for detections of BECN1 immunoreactivity. Rhodamine-conjugated AffiniPure goat anti-mouse IgG (1: 200, #115-025-003, Jackson ImmunoResearch Laboratories) was used to recognize the primary antibodies. For nucleus labeling, the sections were washed with 0.1 M PBS for 30 min and incubated with DAPI (Sigma chemical CO., St. Louis, USA) at room temperature for 5 min. TUNEL solution containing FITC-dUTP (*In Situ* Cell Death Detection Kit, Cat. No. 12 156 792 001, Roche Diagnostics) was used for the detection of DNA fragmentation. Autophagic cell death was identified by the TUNEL(+) BECN1 immunoreactive cells with round (intact) nuclei^54^. Finally, the sections were washed in 0.1 M PBS for 10 min and mounted with Fluorescent Mounting Medium (Dako Cytomation) for image acquirement under a TissueFAXS^**®**^ cytometer (TissueGnostics).

### Automated characterization and quantification of TUNEL(+) BECN1 immunoreactive cells

Images were captured with a fluorescent scanning microscope, followed by an *in situ* quantification using microscopy-based multicolor tissue cytometry (MMTC). In brief, tissue sections were scanned using a TissueFAXS® cytometer (TissueGnostics, Vienna, Austria). Images of the entire tissue sections were captured at 20× amplification. Using the system’s built-in autofocus, the field of striatum was figured-out to estimate the area of the striatum. Analysis of immunofluorescent tissue sections was acquired by the TissueQuest® analysis software (TissueGnostics) that identified TUNEL(+) BECN1 immunoreactive cells using a proprietary search algorithm that first identified DAPI-labelled nuclei and then measured the mean relative fluorescence intensity around each nucleus. The mean relative fluorescent intensity turned out to be the best parameter to visualize BECN1 immunoreactive populations and was used throughout the whole study to distinguish positive and negative cell populations. The fluorescence reactivity determined by TissueQuest® for the negative control samples defined the level of unspecific staining reactivity and allowed determination of the individual cut-off value for each sample. Only objects exceeding the fluorescence intensity in the respective channel were accepted as specifically stained and therefore as marker-positive cells. The amount of DAPI(+) nuclei and the number of TUNEL(+)or BECN1 immunoreactive cells for each sample were analyzed simultaneously^55^.

### Statistical analysis

FC-induced injury in the striata from males and females was compared using two-way ANOVA followed by a Scheffé *post hoc* test. Data on the effects of ho-1 knockout on FC-induced injury severity, histological lesions, behavioral deficits, autophagy and autophagic cell death were analyzed using a multi-way ANOVA to determine the effect of each factor and the interaction between two factors. Significance was accepted at p < 0.05.

## Additional Information

**How to cite this article**: Wang, L.-F. *et al*. Knockout of *ho-1* protects the striatum from ferrous iron-induced injury in a male-specific manner in mice. *Sci. Rep*. **6**, 26358; doi: 10.1038/srep26358 (2016).

## Supplementary Material

Supplementary Information
