# Supplementary material for: Knockout of ho-1 protects the striatum from ferrous iron-induced injury in a male-specific manner in mice
Source: Sci Rep. 2016 May 20;6:26358. doi: 10.1038/srep26358 (PMC4873828; doi:10.1038/srep26358)
Supplement: Supplementary Information [file srep26358-s1.pdf]

Knockout of *ho-1* protects the striatum from ferrous iron-induced injury in a male-specific manner in mice

[1]Li-Fang Wang PhD, [2]Kazunari K. Yokoyama, [3]Chih-Lung Lin, [4]Tzu-Yin Chen M.S., [4]Hsiu-Wen Hsiao M.S., [4]Pei-Chi Chiang M.S., [2,4]Chin Hsu PhD

Supplementary information

S1

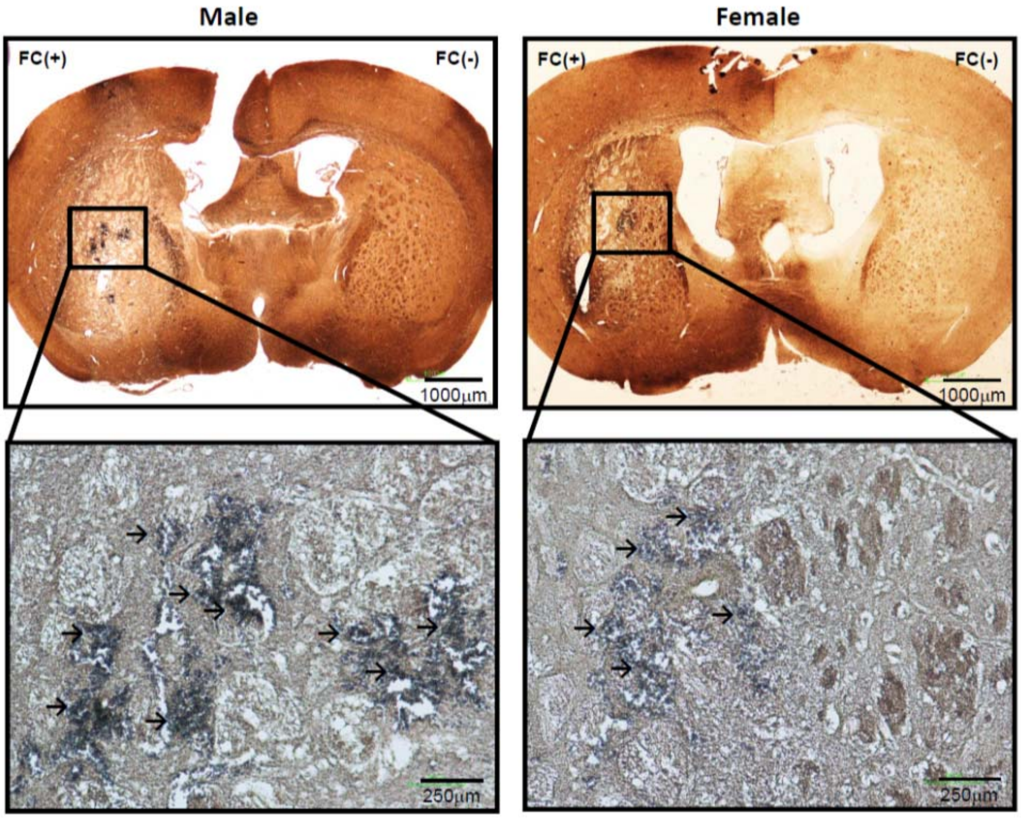

S1 Fig. The Prussian blue assay on brain sections from male and female mice infused with ferrous citrate (FC) in striatum. Two days after FC infusion, the brain tissue containing striatum was sectioned and stained by working solution which is a mixture of equal parts of hydrochloric acid and potassium ferrocyanide prepared immediately before use. Arrows in the lower panels indicate dark blue deposits that confirmed the iron accumulation after FC infusion.

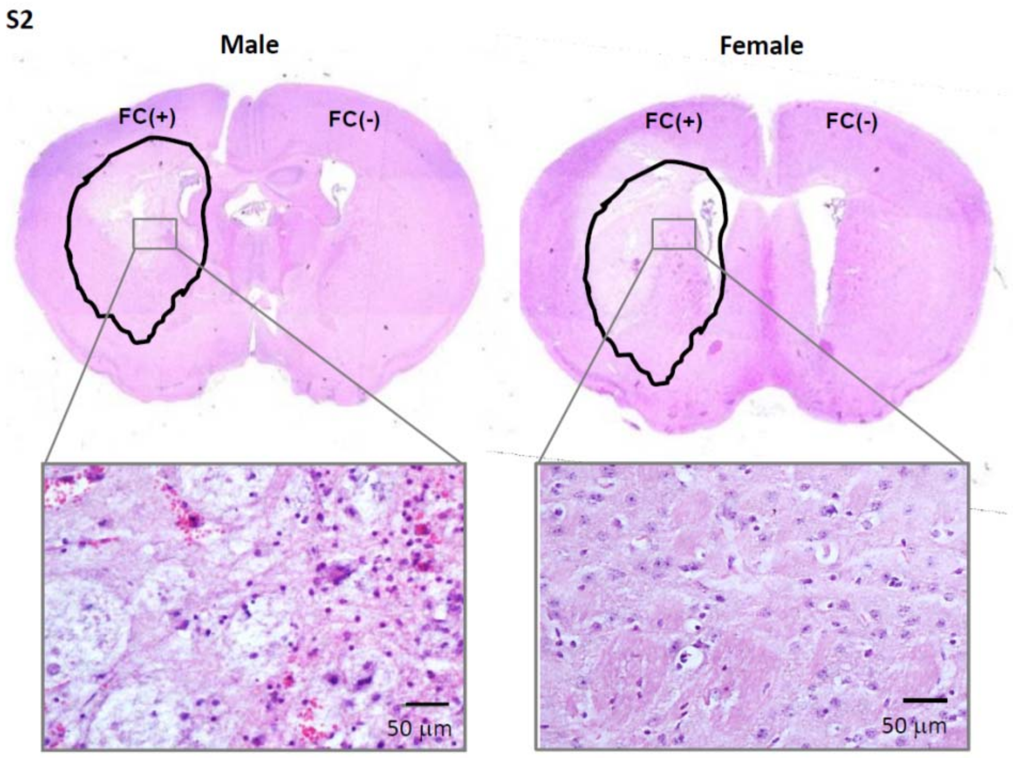

S2 Fig. Histological lesions in striata of male and female mice after FC infusion. The paraffin-embedded tissues were serially sectioned into 10-μm

thick slices. After hematoxylin and eosin (HE) staining, the extents of the histological lesions were analyzed using Image-proPlus according to the staining intensity in the enclosed area of the upper panel. The ratio of ipsilateral hemispheric volume of the striatum to the contralateral hemispheric volume served as an index of histological lesion. The images at the lower panel indicate the higher magnification of the histological injury in the square area of the upper panel is more obvious in male than in female. The scale bars represent the magnifications under microscope.
